# Salivary Levels of IL-6 and IL-17 Could Be an Indicator of Disease Severity in Patients with Calculus Associated Chronic Periodontitis

**DOI:** 10.1155/2018/8531961

**Published:** 2018-02-18

**Authors:** Husniah Batool, Ahmed Nadeem, Muhammad Kashif, Faheem Shahzad, Romeeza Tahir, Nadeem Afzal

**Affiliations:** ^1^Nishtar Dental College, Multan, Pakistan; ^2^Department of Immunology, University of Health Sciences, Lahore, Pakistan; ^3^Department of Oral Pathology, BAMDC, Multan, Pakistan

## Abstract

*Background/Purpose. *Chronic periodontitis is an inflammatory disease of gums that causes loss of supporting structures of teeth, that is, gingiva, periodontal ligament, cementum, and alveolar bone. Levels of various cytokines in the serum, gingival tissues, and gingival crevicular fluid in patients with chronic periodontitis have been studied, but limited data are available on the level of cytokines in saliva. Therefore, a study was designed to determine levels of salivary IL-6 and IL-17 in patients with calculus associated chronic periodontitis.* Materials and Methods.* It was a comparative, cross-sectional study that is comprised of 41 healthy controls and 41 calculus associated chronic periodontitis patients (CP patients). According to the degree of attachment loss, CP patients were subcategorized as mild (CAL 1-2 mm), moderate (CAL 3-4 mm), and severe (CAL > 5 mm) forms of periodontitis. Salivary levels of IL-6 and IL-17 were determined using enzyme-linked immunosorbent assay (ELISA) technique. Data was analyzed using SPSS 20.0.* Results.* Between healthy controls and CP patients (moderate and severe disease), a statistically significant difference was observed in the concentrations of IL-6 and IL-17. In CP patients, the highest mean ± SD of salivary IL-6 and IL-17 was observed in severe CP, followed by moderate and mild CP. Regarding level of IL-6, a statistically significant difference was observed between mild and severe disease and between moderate and severe subcategories of CP patients. Similarly, statistically significant difference was observed in the level of IL-17 between mild and moderate, mild and severe disease, and moderate and severe disease.* Conclusion.* The levels of salivary IL-6 and IL-17 were increased significantly in calculus associated CP patients as compared to healthy controls and these levels increased with the progression of CP.* Clinical Significance.* Salivary levels of IL-6 and IL-17 may help in the subcategorization of CP.

## 1. Introduction

Periodontal diseases are progressive and destructive inflammatory conditions that involve tooth supporting structures like gingiva, periodontal ligament, cementum, and alveolar bone. There are multiple risk factors for this disorder such as bacteria, host response, and genetic predisposition. It can manifest as localized or generalized, that is, involving single tooth or many teeth, respectively [1].

Chronic periodontitis (CP) leads to devastation of supporting structures of teeth, alveolar bone, periodontal ligament, pocket formation, gum recession, and eventually tooth loss. It can occur at any age but is more common in adults and is associated with dental plaque and calculus. Dental fillings, artificial crown, and diseases like diabetes may lead to disease progression [2].

Dental plaque can be supragingival or subgingival and it contains proliferating microorganisms, epithelial cells, leukocytes, and macrophages [1]. Subgingival plaque extends into periodontal pocket and often contains Gram-negative organisms. Therefore, it is responsible for calculus formation, root caries, and slow progressive periodontal disease, while unattached plaque is responsible for periodontal destruction. Mineralization of dental plaque results in dental calculus [3].

Oral cavity is colonized by more than 600 species of microorganisms, but only a few of them are pathogenic and cause disease. Most of these microorganisms are Gram negative, for example,* P. gingivalis, Treponema denticola, and Tannerellaforsythia *with different virulence factors such as lipopolysaccharides (LPS) that induce host immune response by activating multiple cell signaling cascades via Toll-like receptors (TLRs), which may trigger CP [4]. The progression of periodontal inflammation is associated with an increase in microorganisms in subgingival plaque [5, 6].

In periodontitis, microbial pathogens increase inflammatory infiltrate, that is, T-cells, B-cells, macrophages, and neutrophils with concomitant increase in inflammatory cytokines like IL-1, IL-11, IL-6, TNF-*β*, TNF-*α*, TGF-*β*, kinins, and thrombin [7]. In chronic inflammation, proinflammatory cytokines like IL-1, TNF-*γ*, IFN-*α*, and IL-6 play significant role in bone resorption by activating osteoclasts [8–10].

Interleukin-17 (IL-17) is a proinflammatory cytokine secreted by Th-17 cells. It is a powerful activator of neutrophils as it regulates G-CSF and its receptor and chemokine expression [11]. It contributes in the pathogenesis of various autoimmune and inflammatory diseases [12, 13]. It regulates antimicrobial activity of molecules like calgranulins, *β*-defensins, and mucin [11]. Its increased level has been documented in CP [14, 15]. Although periodontal infection* (P. gingivalis)* induces IL-17, the protective role of IL-17 against bone destruction has also been suggested [12, 16].

IL-6 is produced by many cells in response to LPS and it has both proinflammatory and anti-inflammatory roles. It is involved in inflammatory, regenerative, metabolic, and neural processes [17]. In CP, increased level of IL-6 in gingival crevicular fluid [18] and its significant reduction in serum after nonsurgical treatment of CP has been reported [19]. However, no significant differences in the levels of various cytokines in saliva of CP patients and healthy individuals were suggested [20].

In CP, most of the studies have investigated level of cytokines in serum or gingival crevicular fluid. The current study was designed to determine level of IL-6 and IL-17 in the saliva of patients with calculus associated CP.

## 2. Materials and Methods

This comparative, cross-sectional study was carried out in the Department of Immunology, University of Health Sciences (UHS), Lahore, after approval by Ethical Review Committee of UHS. Healthy individuals as controls and calculus associated CP patients from 18 to 55 years were recruited from Punjab Dental Hospital Lahore and Sheikh Zaid Hospital Lahore. Forty-one healthy controls and the same number of calculus associated CP patients were included. Pregnant females and patients with malignancy, autoimmunity, infectious disease, edentulous, xerostomia, or traumatic oral ulcers, having prosthesis or dentures, were excluded. Patients with periodontitis other than calculus associated CP, for example, aggressive periodontitis, periodontitis associated with systemic diseases, and necrotizing periodontitis, were excluded. After an informed consent was obtained, 3–5 ml of saliva was collected from each subject as suggested by Prakasam and Srinivasan (2014) [21] that was transported in ice bag to the Department of Immunology, UHS, Lahore.

Periodontal examination of each subject was performed by the consultant dental surgeon. For the selected teeth, probing depth, clinical attachment loss, and bleeding on probing were recorded. CP patients with minimum ten natural teeth, attachment loss of ≥1 mm in >30% of the sites examined, presence of abundant calculus, and radiographic confirmation of bone loss were included. Stages of CP were determined on the basis of criteria used by Wiebe and Putnins (2000) [22]. On the basis of clinical attachment loss (CAL), CP patients were subcategorized as mild (CAL 1-2 mm) (10 patients), moderate (CAL 3-4 mm) (16 patients), and severe (CAL > 5 mm) (15 patients) [22]. The samples were centrifuged at 1217.7*g* for 10 minutes and supernatant containing purified saliva was separated and kept at −80°C until cytokines were estimated by ELISA technique [21].

## 3. Statistical Analysis

The data was entered and analyzed using IBM SPSS 20.0. Mean ± SD was calculated for quantitative variables and qualitative variables were expressed as frequencies, percentages, and graphs. Kruskal-Walis test to normally distributed data and Post Hoc Tukey test to observe group means differences were applied. A *p* value of ≤0.05 was considered as statistically significant.

## 4. Results

Highest mean ± SD of age was observed in CP patients compared to healthy controls and on comparison, there was no statistically significant difference between healthy controls and CP patients (*p* = 0.07). Demographic data of the studied subjects is presented in Table 1. Mean ± SD of IL-6 and IL-17 was high in CP patients compared to healthy controls with a statistically significant difference (*p* < 0.001 in each) (Table 2).

Mean ± SD of IL-6 and IL-17 was highest in the severe periodontitis followed by moderate and mild CP, while the lowest concentration was in healthy controls. An increase of mean ± SD of IL-6 and IL-17 was observed with the progression of disease, that is, from mild to severe CP (Table 3).

While comparing the levels of IL-6 in healthy subjects with the subcategories of CP patients, a statistically significant difference was observed with moderate and severe form of CP (*p* = 0.002 and <0.001, resp.) (Table 3). Regarding levels of IL-17, a statistically significant difference was observed between healthy subjects and CP patients with moderate and severe form of disease (*p* = 0.001) (Table 3)

## 5. Discussion

CP represents long lasting inflammation of periodontal tissues where microbes are a major etiological factor and the hallmark of periodontal diseases is bone loss. Virulence factors produced by* P. gingivalis* such as Arg- and Lys-gingipain proteinases are key factors for host tissue invasion which leads to activation of immune-inflammatory processes. Subsequently, various molecules (proteases, MMPs, cytokines, etc.) are activated leading to destruction of connective tissue attachment and alveolar bone loss [4]. It has been documented that, in humans, production of IL-6 and matrix metalloproteinase-1 (MMP-1) is upregulated by periodontal ligament (PDL) fibroblasts. Therefore, IL-17 induces production of MMP-1 both directly and indirectly by increasing IL-6 production, thus resulting in the destruction of collagens in the periodontal ligament [23].

In the current study, female predominance was observed and it is in agreement with previous studies where researchers investigated the relationship between proinflammatory cytokines in saliva and periodontal status [24]. Mean ± SD of age of CP patients was high compared to healthy controls which is not in concordance with the Romanian study that reported high mean ± SD of age of healthy individuals compared to CP patients that could be due to the age range of the patients as they included > than 18 years of age, while in the current study adults were enrolled [25].

Th17 cell differentiation and expansion is directed by IL-6 and other cytokines. IL-17A, the proinflammatory cytokine secreted by the activated Th17, has been involved in a number of human autoimmune and inflammatory diseases [26]. It also regulates molecules with direct antimicrobial activity like calgranulins, *β*-defensins, and mucin [11]. It has been reported that IL-17 is involved in bone destruction by inducing of RANKL production [27]. Many studies assessed the role of IL-17 in chronic periodontitis but still it is an open question whether IL-17 stimulates bone resorption or protects bone in chronic periodontitis although increasing evidence indicates increased IL-17 expression in both chronic and aggressive periodontitis [28]. In the present study, mean ± SD of IL-17 in saliva was high in CP compared to healthy controls and it gradually increased with the severity of disease. Corroborating our data, previous studies have documented increased level of IL-17A in saliva, serum, and gingival crevicular fluid (GCF) in periodontitis compared to healthy subjects and also reported important role of IL-17 in gingival inflammation and bone loss [21, 29–32].

Besides the destructive role of IL-17 in chronic periodontitis, the protective role of IL-17 against pathogen associated bone destruction has also been reported. IL-17 enhances bone destruction in rheumatoid arthritis, but IL-17 signaling through IL-17 receptor exerts bone protective effect on periodontal bone loss because it provides antimicrobial defense against periodontal organisms like* P. gingivalis *[12] and decreases alveolar bone resorption due to defective neutrophil chemotaxis [33].

IL-6 is involved in antigen-specific immune responses and inflammatory reactions. IL-6, CXCL-8, and TNF-*α* may have an important role in the pathogenesis of chronic periodontitis and detection of these cytokines may be beneficial to identify periodontitis patients. IL-6 along with IL-1*β* and IL-23 is required for the initiation, amplification, and stabilization stages of Th17 cell differentiation [25].

In the current study, salivary IL-6 was high in CP compared to healthy controls. Studies have reported similar results [18, 34–36]. The integrity of bone tissue depends on the balance between bone formation by osteoblasts and bone resorption by osteoclasts. Therefore, several proinflammatory cytokines were identified as key molecules contributing to the destruction of periodontal tissues, like IL-1, TNF-*α*, IFN-*α*, and IL-6. Thus, it is possible that high level of IL-6 in the patients may have acted as a osteoclastogenic factor by inducing the local osteoclast differentiation that could terminate in bone resorption. IL-6 induced by IL-17F or IL-17A may play a potential feedback loop in the pathogenesis of chronic periodontitis. However, whether it is a positive or negative feedback loop, it is still arguable [37].

## 6. Conclusion

Salivary levels of IL-6 and IL-17 were significantly higher in patients with calculus associated CP compared to healthy subjects. These cytokines increased as the disease progressed from mild to moderate and severe form. Therefore, we can conclude that the salivary level of IL-6 and IL-17 may help in the subcategorization of periodontitis.

## Figures and Tables

**Table 1:** Number, percentage, mean ± SD, and comparison of gender and age between two groups.

Variables	Healthy subjects (*n* = 41)	CP patients (*n* = 41)	*p* value
Gender			
M *n* (%)	18 (44%)	17 (42%)	1.00
F *n* (%)	23 (56%)	24 (58%)	
Age (mean ± SD)	26.59 ± 3.59	38.26 ± 12.06	0.07

Statistically significant *p* ≤ 0.05, CP = chronic periodontitis, M = male, F = female, and *n* = number.

**Table 2:** Comparison of IL-6 and IL-17 levels between healthy controls and CP patients.

Variables	Healthy controls (Mean ± SD)	CP patients (Mean ± SD)	*p* value
IL-17 (pg/ml)	1.21 ± 0.92	9.56 ± 7.83	<0.001
IL-6 (ng/l)	16.87 ± 9.34	73.03 ± 57.02	<0.001

Statistically significant *p* ≤ 0.05; CP = chronic periodontitis.

**Table 3:** Levels of IL-6 and IL-17 in CP patients and healthy controls.

Variables	Healthy controls (41) (Mean ± SD)	Mild CP (10) (Mean ± SD)	Moderate CP (16) (Mean ± SD)	Severe CP (15) (Mean ± SD)	*p* value
IL-17 (pg/ml)	1.21 ± 0.93	2.54 ± 1.13	6.68 ± 2.33	17.34 ± 1.98	0.705^1^, 0.001^2^, 0.001^3^, 0.023^4^, 0.001^5^, 0.001^6^
IL-6 (ng/L)	16.87 ± 9.34	39.27 ± 16.47	56.43 ± 10.77	113.23 ± 20.21	0.265^1^, 0.002^2^, <0.001^3^, 0.610^4^, <0.001^5^, <0.001^6^

Statistically significant *p* ≤ 0.05, CP = chronic periodontitis, ^1^Healthy and mild, ^2^Healthy and moderate, ^3^Healthy and severe, ^4^Mild and moderate, ^5^Mild and severe, ^6^ Moderate and severe.
